# Photoinduced Carbonyl Radical Luminescence in Host–Guest Systems

**DOI:** 10.1021/acsami.3c14730

**Published:** 2023-12-12

**Authors:** Juanjuan Liu, Huajie Yu, Farhan Siddique, Glib V. Baryshnikov, Hongwei Wu

**Affiliations:** † Key Lab of Science and Technology of Eco-Textile, Ministry of Education, National Engineering Research Center for Dyeing and Finishing of Textiles, College of Chemistry, Chemistry and Chemical Engineering, Donghua University, Shanghai 201620, China; ‡ Laboratory of Organic Electronics, Department of Science and Technology, Linköping University, SE-60174 Norrköping, Sweden

**Keywords:** radical emission, host−guest coassembly, fluorescence, stimuli-responsive property, ammonia compound identification

## Abstract

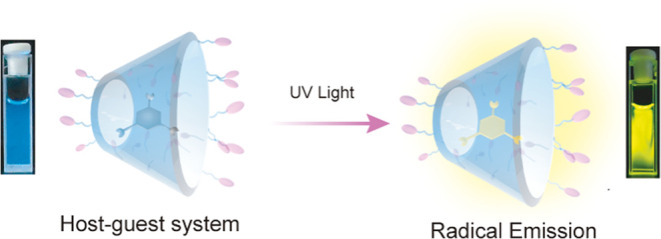

Developing a free radical emission system in different states, especially in water, is highly challenging and desired. Herein, a host–guest coassembly strategy was used to protect the in situ photoactivated radical emission of carbonyl compounds in solid and aqueous solutions by doping them into a series of small molecules with hydroxyl groups. The intermolecular interactions between host and guest and the electron-donating ability of the hydroxyl group can significantly promote the formation and stabilization of luminescence by carbonyl radicals. Accordingly, the stimuli-responsive property of the free radical system was investigated in detail, and the self-assembled aggregates showed photoactive and thermoresponsive behaviors. In addition, an advanced ammonia compound identification system can be built based on a radical emission system. Our design strategy sheds light on developing free radical systems that can emit in various states, which will greatly broaden the application range of free radicals.

## Introduction

Recently, free radicals with special properties such as magnetism, electrical conductivity, photoluminescence, etc. have attracted considerable attention.^1−11^ Triphenyl methyl (TM) radical derivatives are the most studied free radical emitter materials with fascinating luminescent properties^12,13^ and have shown high external quantum efficiency in OLEDs.^11,14^ In addition, the free radical systems with multistimulus response properties have demonstrated many potential and practical applications.^15−17^ The TM radical derivative has exhibited temperature-responding emission at low temperatures under the magnetic field control.^5,16,18^ Our group reported the photoactivated and thermoresponsive radical emission of carbonyl compounds in polymers and ion liquids.^19^ Based on these studies, free radical materials are expected to become the next generation of new luminescent systems.

However, organic materials with free radical emission properties are relatively rare due to the fact that they are chemically reactive, and their emission can be quenched easily by air, solvents, and water. Generally, a large steric hindrance group or extended conjugation was needed to protect their emissions. Unfortunately, the modification of large steric hindrance groups or extension of conjugated structures requires a tedious synthesis process, which significantly impedes the development and practicality of free radical materials. Nowadays, researchers have developed a series of simple supramolecular strategies including host–guest coassembling,^20^ polymer doping,^21^ and crystal environment to restrict the nonradiative transitions of radical excitons.^6,15,17,22^ Especially in the host–guest systems, the guest radicals dispersed in the host medium avoid recombination with each other. Nishihara et al. found that (3,5-dichloro-4-pyridyl)bis(2,4,6-trichlorophenyl)methyl (**PyBTM**) radicals can show monomer and excimer emission by changing the doping concentration of host molecule.^6^ However, so far, the number of studies using host–guest doping to protect radical emission is still very limited, and there is a lack of research on doping them into hydrophilic hosts for obtaining the hydrophilic free radical emitting material. Therefore, doping of the radical molecules in commercially accessible hydrophilic hosts is highly desired.

**Figure 1:**
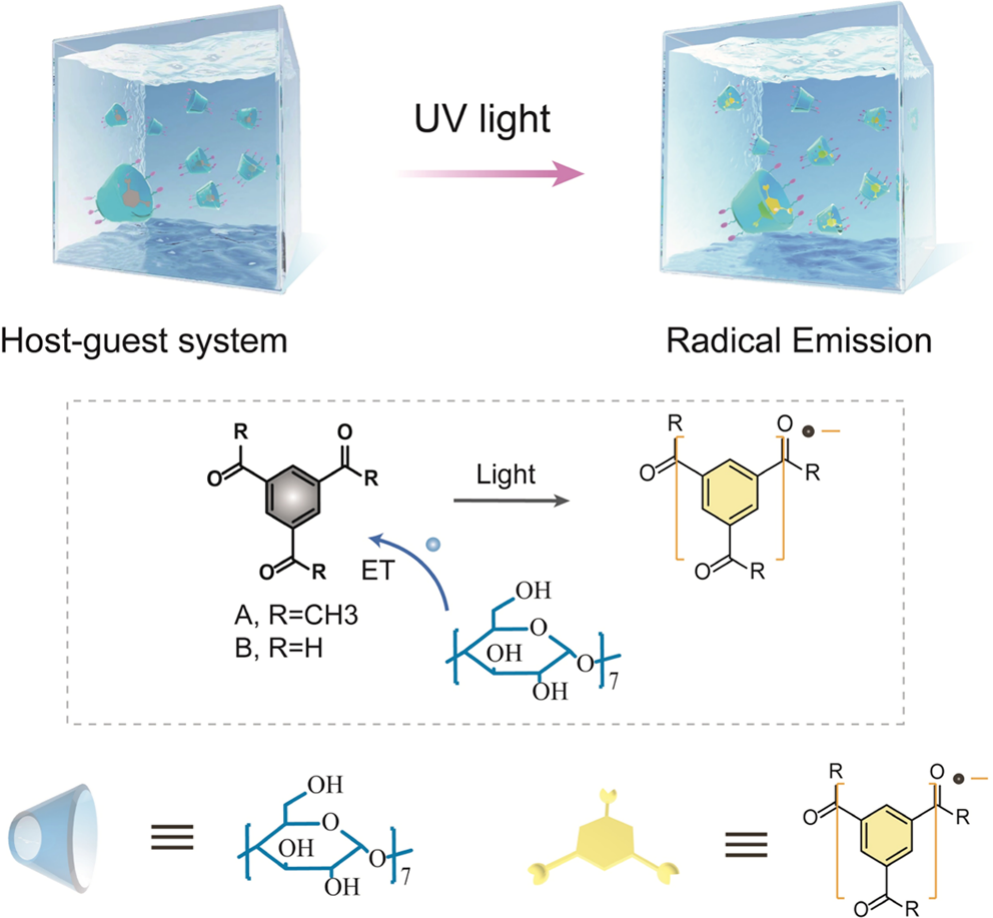
Illustration of photoinduced radical emission of coassembly systems taking **α-CD** as an example. The coassembly system is formed by host–guest interactions between carbonyl compounds and **α-CD** in water. Then, the electron transfer between **α-CD** and carbonyl compounds can induce the formation of carbonyl free radicals after light irradiation, and the host–guest interactions can facilitate the radical emission effectively.

Herein, the coassembly system of carbonyl guest compounds (Figure 1) and hydroxyl-containing α-cyclodextrin (**α-CD**) molecules was built in solid powder and water, respectively. It is beneficial that hydroxyl-containing molecules can stabilize the carbonyl free radicals by intermolecular interactions, and the hydroxyl groups of the host can transfer electrons to the guest carbonyl group to facilitate the formation of radicals after light irradiation.^19,23,24^ Therefore, we first doped carbonyl compounds into polyhydroxy compounds to obtain solid crystalline powders in which the nonradiative transfer of radical excitons can be largely limited, leading to the apparent solid-state free radical emission after light irradiation. On the other hand, the hydrophilic host should be required to induce strong intermolecular interactions between host and guest in water. For example, cyclodextrins with many hydroxyls can form intermolecular interactions with guest molecules to promote or tune the emission of the molecules in an aqueous solution^25−27^ Hence, hydrophilic compounds such as cyclodextrin, trehalose, sucrose, and F127 were used to coassemble with carbonyl compounds to induce the free radical emission in water after light irradiation (Figure 1). In addition, the obtained radical emission system can be used to build advanced systems for the detection of ammonia/amine compounds based on the intermolecular interaction or reaction between carbonyl radicals and ammonia/amine species.

## Results and Discussion

**Figure 2:**
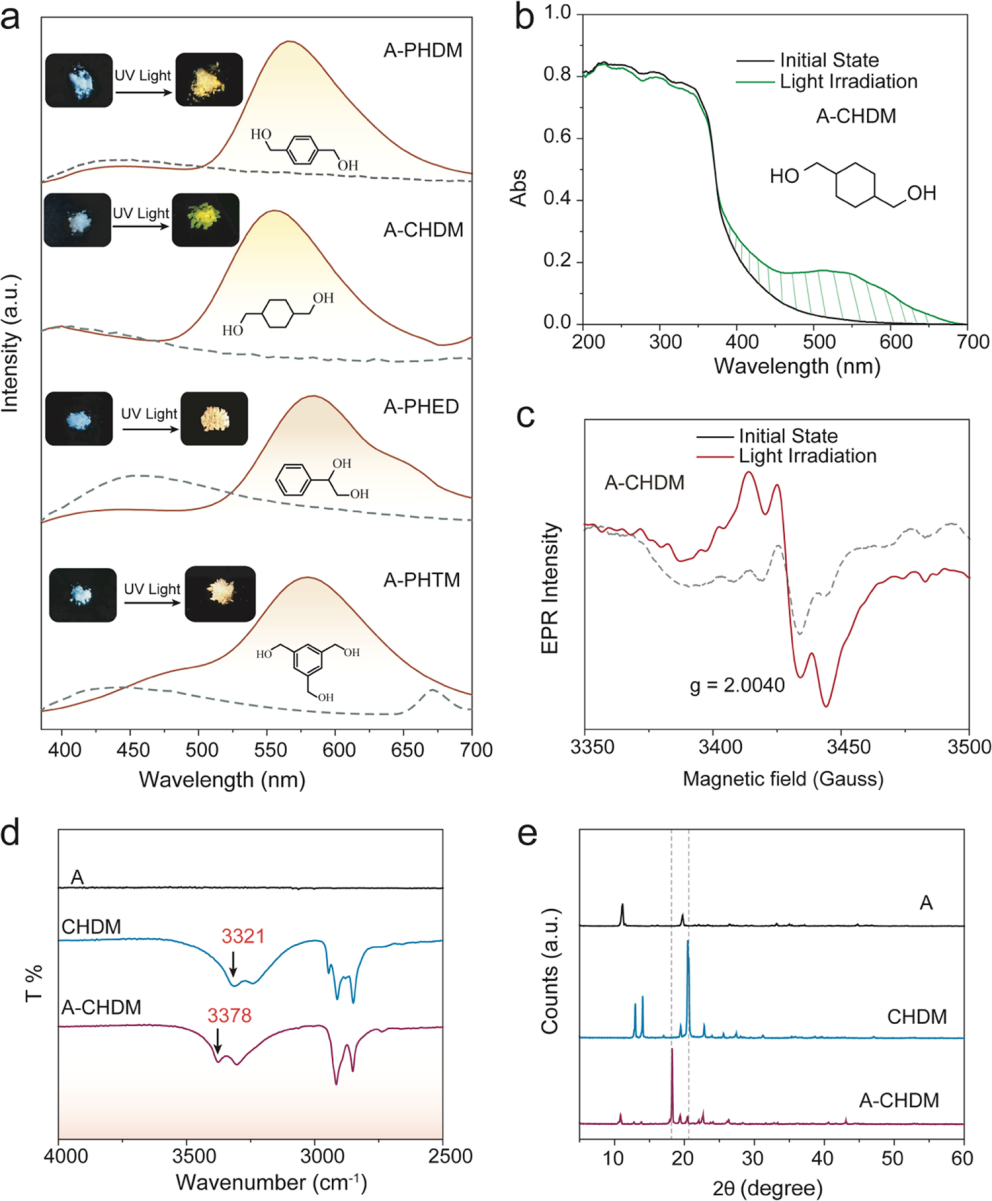
(a) Fluorescence emission spectra of **A** with four molecules before and after 365 nm UV light (*P* = 10 W) irradiation. Inset: the luminescent photographs of the systems before and after UV irradiation. (b) Absorbance spectra of **A-CHDM** at the original state and after 365 nm light irradiation. (c) EPR spectra of **A-CHDM** (1:1 wt %) in the powder state. (Note that this high concentration was chosen because it can generate more radical species for EPR testing. Also, the initial weak EPR peak was caused by instrument excitation light in the test process.) (d) FTIR spectra of compounds **A**, **CHDM**, and **A-CHDM** (1:10 wt %). (e) Powder XRD patterns of **A**, **CHDM**, and **A-CHDM** (1:10 wt %).

Four polyhydroxy compounds (**CHDM**, **PHDM**, **PHED**, and **PHTM**) were used as host molecules to dope with carbonyl compound guests (**A** or **B**) in organic solvents. The solid powders were obtained by evaporating the solvent slowly. These powders did not show the emission initially, but the yellow or orange luminescence was exhibited after light irradiation by a 365 nm ultraviolet (UV) light (*P* = 10 W) for 10 s (Figure 2a). We should state that the light source of the fluorescence spectrophotometer can photoactively emit radicals in the test process, such that in some cases, the initial state sample also shows a similar but very weak emission with the radical emission. The stoichiometry between host and guest molecules in the binary complex was determined as 10:1 or 100:1 (host/guest, weight ratio (wt %)) by comparing the emission intensities (Figure S1). Excessive guest ratio led to weak emission due to aggregation-caused quenching (ACQ). This photoinduced emissive process is similar to our previous dye–PVA system in which the yellow radical emission was observed after light irradiation.^19^ These powders showed the maximum emission wavelength ranging from 550 to 580 nm after a 365 nm UV light irradiation (Figure 2a). The host molecules with the electron-donating benzene ring induced more red-shift emission compared to aliphatic compounds, which should be due to the formation of charge transport states with carbonyl compounds (Figures 2a,b and S2). It is noted that there are no changes in the absorption spectra after UV light irradiation for different individual host materials, which excludes the possibility of radical emission from host molecules (Figure S3).

In addition, these systems showed nanosecond-level emission lifetimes, which rule out the possibility that the luminescence involves the high-spin state (Figure S4). Meanwhile, the UV spectra also showed the obvious red-shift absorbance after the irradiation (Figure 2b). A new absorption peak that appears at 500 nm of the **A-CHDM** is similar to our previous report on the absorbance of the free radical.^19^ Therefore, in order to prove the generation of the light irradiation-induced radicals, we collected the electron paramagnetic resonance (EPR) spectra of the **A-CHDM** sample, and a significant free radical signal was found after light irradiation (Figure 2c), indicating the emergence of radicals, and the *g* value of **A-CHDM** radical was estimated as 2.0040. The ^1^H NMR study was also carried out to confirm the emission species, and there is no variation in the chemical shift (Figure S5). Meanwhile, the HPLC chromatogram showed that no new peaks were found before and after light irradiation (Figure S6). These results are ascribed to the free radical being difficult to discern by NMR and HPLC.

Due to the fact that the carbonyl group is an electron-withdrawing acceptor group, the transfer of unpaired electrons from the hydroxyl molecules to **A** in solid powder can be expected.^28^ To demonstrate that electron transfer is important for radical emission, the host molecule **BP** without a hydroxyl group was used to assemble with the guest molecules. Generally, the resulting solid systems did not show obvious radical emission, and almost no absorption red-shift was observed after light irradiation, which proved the importance of hydroxyl groups in the doping system for radical emission (Figure S7).

In addition, to protect the excited states of light-emitting molecules, the host should form a strong intermolecular interaction with the guest molecules.^29−31^ The Fourier transform infrared spectra of compounds **A**, **CHDM**, and **A-CHDM** were investigated, and the stretching vibration peak of the hydroxyl group of the host molecule **CHDM** moves from 3321 cm^–1^ (**CHDM**) to 3378 cm^–1^ (**A-CHDM**) (Figure 2d), suggesting the formation of strong intermolecular interactions between host and guest that will facilitate the free radical emission in the powder state.

The X-ray diffraction (XRD) patterns of the **A**, **CHDM**, and **A-CHDM** solid powders were also investigated. The strong diffraction peaks of **A-CHDM** suggest the powder with a well-crystalline nature, which means it can decrease the nonradiation transfer of free radical exciton to promote the emission (Figure 2e). The obvious peak shift could be observed in the **A-CHDM** sample compared with that of **CHDM**, indicating the change in molecular arrangement, which suggests the coassembly of host and guest. To demonstrate the generality of our strategy, compound **B** was also investigated in host–guest systems, and a similar photoinduced emission phenomenon was observed in B-host systems (Figures S8–S10). From these results, we conclude that strong intermolecular interactions could be formed between the carbonyl compounds and polyhydroxy guest compounds.

In the next stage, we focused on the design of a hydrophilic host–guest assembly system. Therefore, according to the above research, α-cyclodextrin (**α-CD**) was chosen to build a rigid environment to protect the excited state of luminescence molecules because it contains a large number of hydroxyl groups. First, **A** and **α-CD** (1:1 wt %) were completely mixed in water by ultrasound to acquire the system. The system did not show obvious free radical emission after light irradiation (Figure S11). This phenomenon may be caused by the poor water-solubility property of compound **A**, resulting in the failure to disperse them into the water and capture them by cyclodextrin.

**Figure 3:**
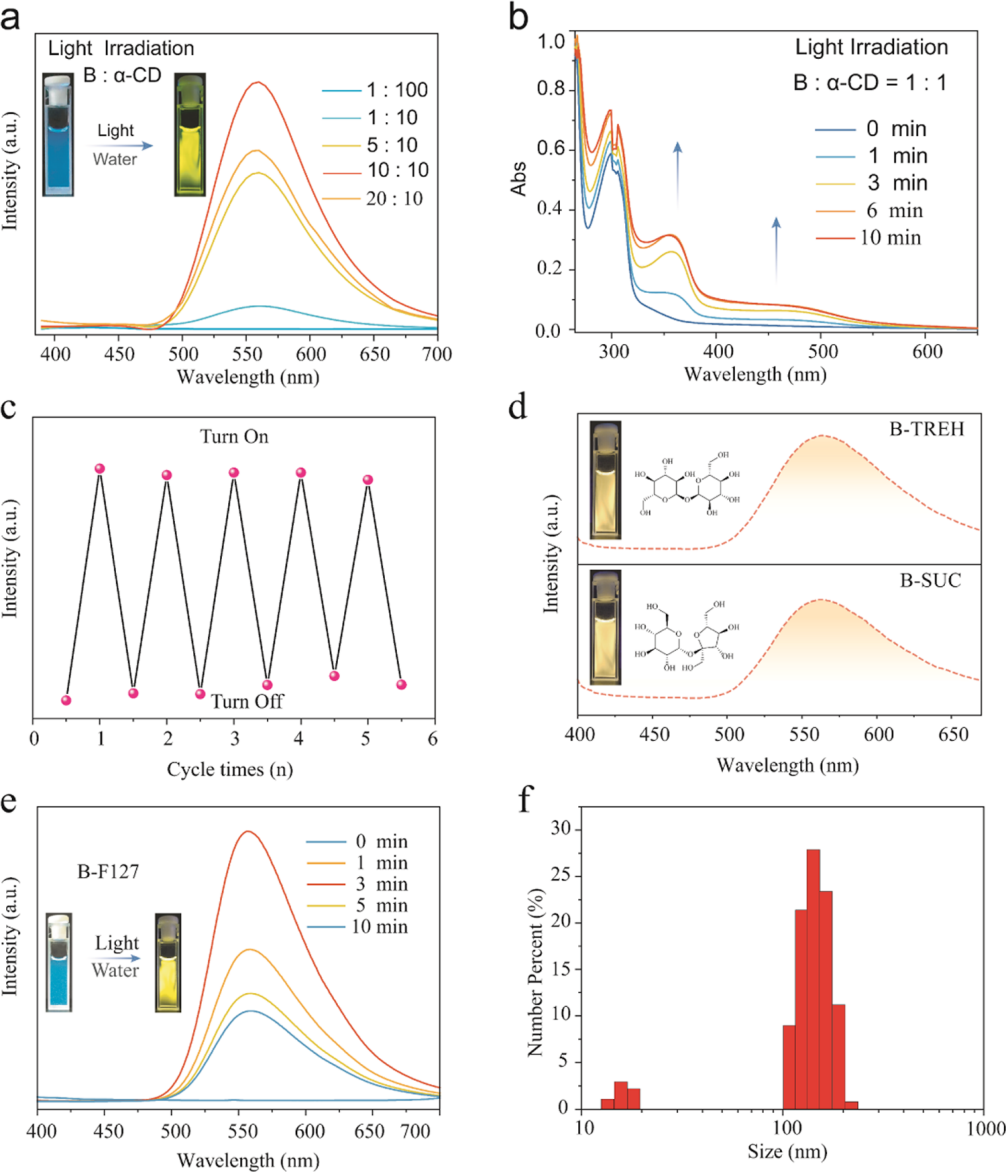
(a) Fluorescence emission spectra of **B-α-CD** at different doping ratios after 365 nm light irradiation. (b) Absorption spectra of **B-α-CD** after different irradiation times. (c) Emission intensity changes of the maximum emissive wavelength of **B-α-CD** at repeated cycles. (d) Emission spectra of **B-TREH** and **B-SUC** (1:1 wt %) after 365 nm light irradiation. (e) Fluorescence spectra of **B–F127** (1:1 wt %) after different light irradiation times. (f) Particle size distribution of **B–F127** (1:1 wt %) measured by DLS.

However, **B** is more hydrophilic than **A**. The **B-α-CD** coassembly system in water exhibited bright yellow emission with the maximum emission peak around 550 nm after 365 nm UV light (*P* = 10 W) irradiation for 10 s (Figures 3a and S12). The new absorbance peak in the visible light region confirmed the formation of free radicals of the **B-α-CD** system in water (Figure 3b). The ^1^H NMR spectra were also measured to confirm the emission species, and there is no obvious variation in the chemical shift (Figure S13). The new signals in the aromatic region found in the NMR spectra of **B-α-CD** should be due to the radical being more active in the water.^32,33^ The emission spectra under different doping concentrations were also studied. With the increase of the concentration of **B**, the luminescence intensity of the system gradually increased, reaching the strongest emission intensity when the doping ratio was 1:1 (Figure 3a). Then, the emission intensity decreased when the content of dopant **B** was high due to the excess aggregation and side reactions can quench the radical emission.^6,19,34−36^ The EPR spectra of **B-α-CD** (1:1 wt %) at different irradiation times were also measured (Figure S14), and the results show that the EPR signal of free radicals decreases after a long time of light irradiation, which indicates that excessive light irradiation can lead to the side reaction of free radicals. The fluorescence lifetime of the **B-α-CD** system in water was fitted to be 3.5 ns (Figure S15), which is similar to our previous report,^19^ proving that the photoinduced emission should be from free radical emission. In addition, the luminescence system has excellent repeatability. The emission of the system can be automatically extinguished (10 min) and again photoactivated by UV light. After more than five cycles of switching, the system still exhibited good luminescence performance (Figure 3c).

Moreover, water-soluble trehalose and sucrose with many hydroxyls were chosen as hydrophilic hosts, and similar yellow radical emissions also appeared in these coassembly systems after light irradiation (Figures 3d, S16, and S17). In addition, a hydrophilic surfactant with an alkoxy chain named **F127** was used to assemble with compound **B**. The bright yellow emission with the largest emission wavelength of 553 nm was generated in the system after 365 nm light irradiation. Also, the best irradiation time is 3 min (Figure 3e). **B–F127** in water showed the nanometer particle size measured by dynamic light scattering (DLS), which proves the successful coassembly of **B** with **F127** (Figure 3f). The free radical emission should be because the alkoxy chain can transfer the electron to **B** for promoting the formation of the free radical of **B**.^19^ These results prove that the hydrophilic guests that can form strong intermolecular interactions with **B** can induce free radical emission of **B** in water.

**Figure 4:**
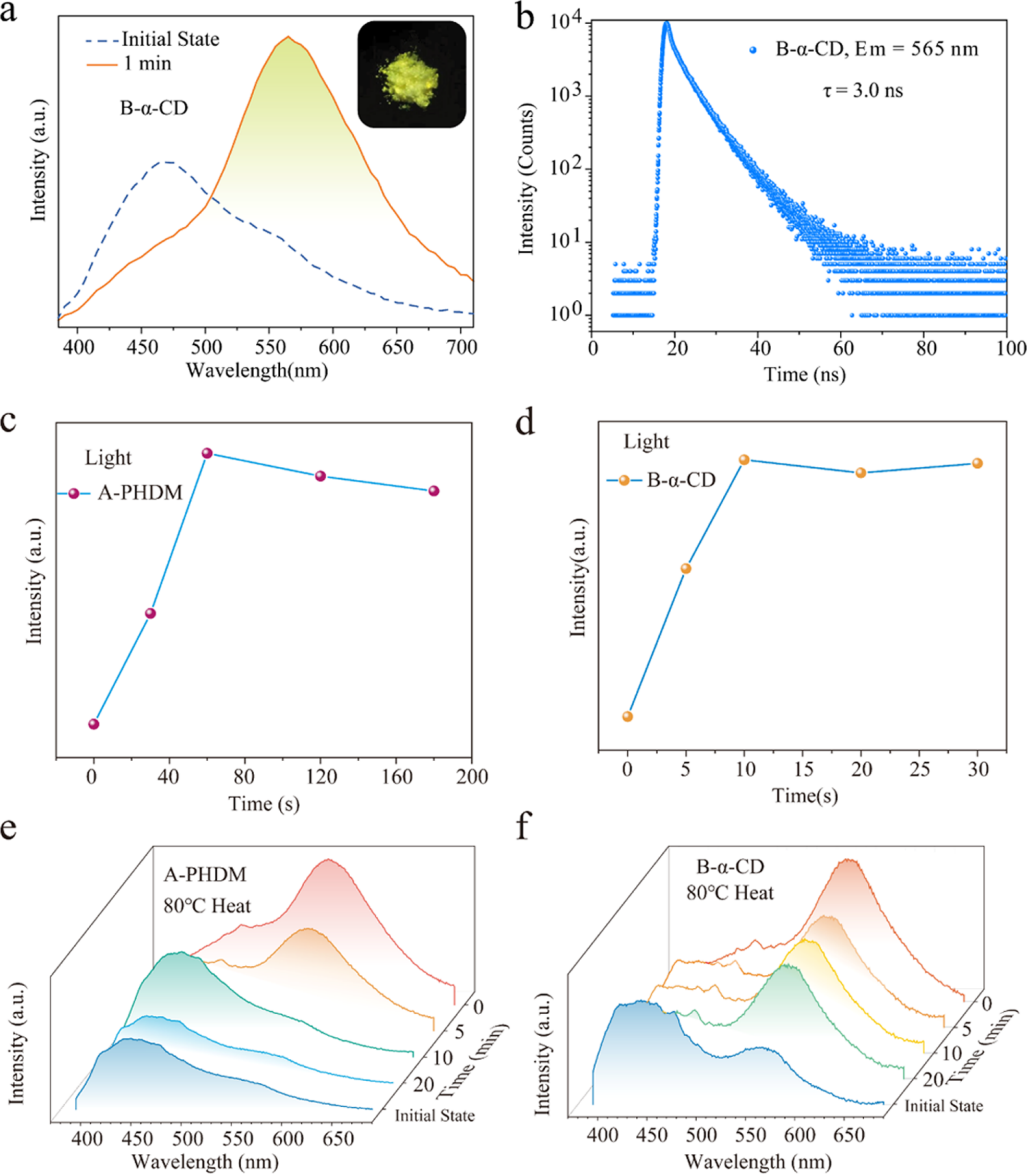
(a) Emission spectra of **α-CD** with **B** (1:1 wt %) in the initial state and after 365 nm light irradiation. (b) Emission lifetime of **α-CD** with compound **B** under 565 nm emission and 365 nm excitation. (c) Fluorescence intensity changes of **A-PHDM** (1:10 wt %) after different irradiation times. (d) Fluorescence intensity changes of **B-α-CD** (1:1 wt %) after different heating times at 80 °C. (e) Fluorescence intensity changes of **A-PHDM** (1:10 wt %) after different heating times at 80 °C. (f) Fluorescence intensity changes of **B-α-CD** (1:1 wt %) after different heating times at 80 °C.

Due to the solid-state rigid environment that can limit the molecular motions well, the solid-state radical emission could be enhanced and more stable compared to the solution state. Indeed, the **B-α-CD** solid powder exhibited a more stable yellow emission than in solution after light irradiation by UV light (Figure 4a). The emission lifetime of the solid powder is 3.0 ns (Figure 4b). We also tested the EPR spectra of **B-α-CD** (1:1 wt %) in the powder state. The free radical signal was not found before UV lamp irradiation, while the new peaks were observed in the lower field after light irradiation, and the *g* value of free radical is 2.0046 (Figure S18), suggesting the formation of free radicals. More interestingly, the obtained **B-α-CD** solid powder showed a different photoactive lifetime and thermoresponsive property compared to the before obtained systems like **A-PHDM**. The **A-PHDM** solid powder needs 30 s of light irradiation time to obtain the obvious free radical emission (Figure 4c). However, it only needs 5 s for the **B-α-CD** solid powder (Figure 4d).

In addition, the photoinduced emission of **A-PHDM** solid powders was weakened when heated at 80 °C for 5 min and largely quenched after 10 min (Figure 4e). In converse, the photoinduced emission of **B-α-CD** solid powder could be weakened by heating at 80 °C for 5 min, but the emission intensity did not change significantly after continuous heating for 10 or even 20 min (Figure 4f). This is probably because the **B-α-CD** system has much stronger intermolecular interactions than the **A-PHDM** system; thus, it can better stabilize the free radical emission. It is noted that **B-TREH** and **B-SUC** solid powder also showed similar solid-state photoinduced emissions (Figures S19 and S20).

**Figure 5:**
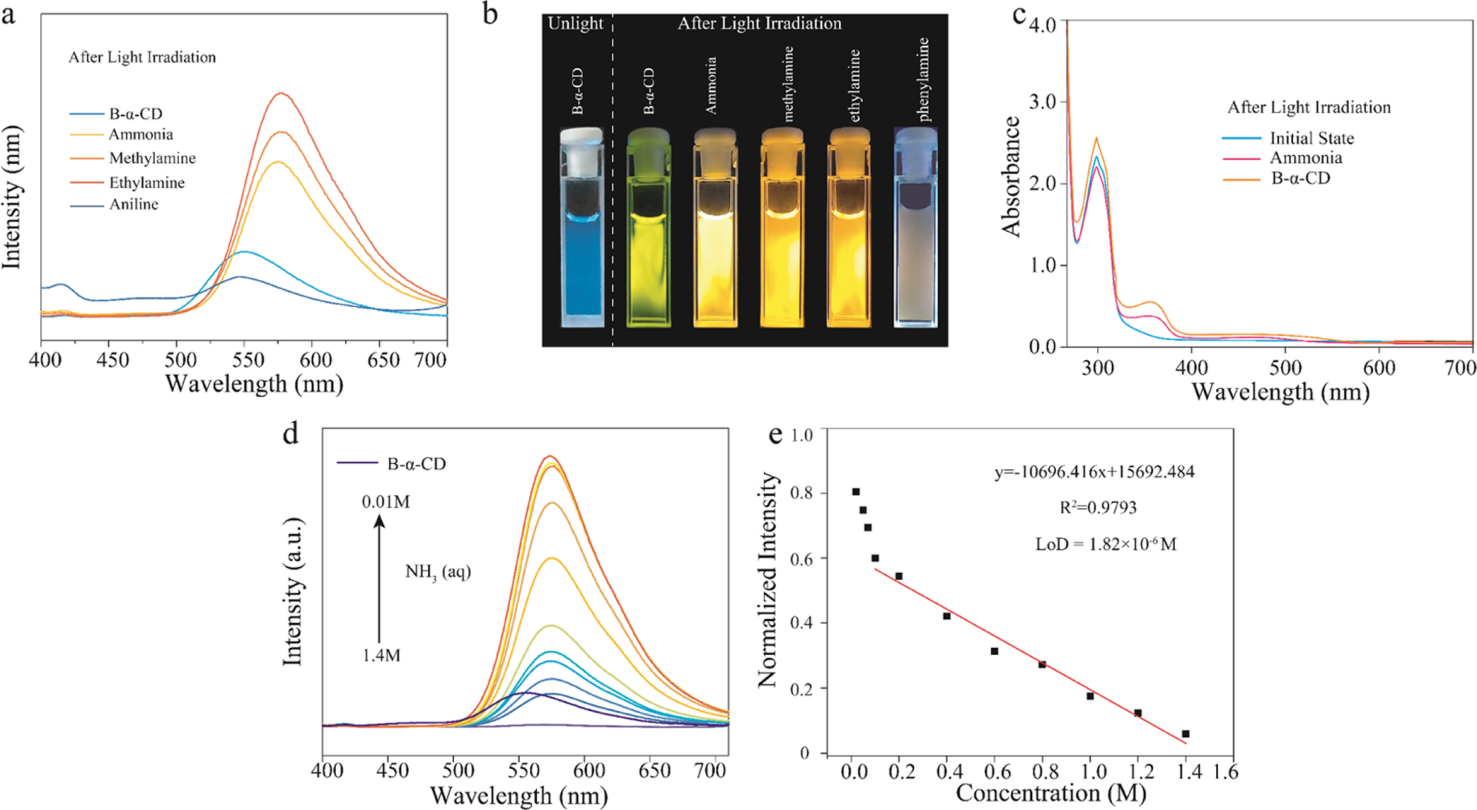
(a) Fluorescence emission spectra of **B-α-CD** (1:1 wt %; *B* = 6 × 10^–3^ M) and **B-α-CD** amine solutions after light irradiation. (b) Luminescence image of the amine-based aqueous solution after light irradiation. (c) Absorbance spectra of the **B-α-CD** ammonia solution at the initial state and under 365 nm light irradiation. (d) Change in the emission of an aqueous dispersion of **B-α-CD** (λ_ex_ = 365 nm) with increasing amounts of NH_3_ (aq). Inset: photographs of an aqueous dispersion of **B-α-CD** under UV lamp irradiation showing the changes in fluorescence after the addition of NH_3_ (aq). (e) Linear fit to the plot of emission against the concentration of NH_3_ (1.4 × 10^–4^–5 × 10^–6^ mol) added to an aqueous dispersion of **B-α-CD** to calculate the limit of detection (LOD) for NH_3_ (aq).

Because the carbonyl and amine groups can interact or react with each other, many carbonyl-containing compounds have been used to detect organic amine molecules in various environments.^37,38^ Inspired by these studies, we selected typical amine molecules commonly used in industry (such as ammonia, methylamine, ethylamine, and aniline) as representative target analytes to detect different ammonia compounds. First, the various ammonia analytes were added to aqueous suspensions of **B-α-CD**, and the resulting fluorescent response spectra were recorded. For the ammonia-added solution, we can visually observe that the luminescence of the ammonia-added solution is enhanced under excitation by the UV lamp (Figure 5a,b). Also, the largest emission peak was red-shifted by 25 nm compared with **the B-α-CD** system (Figure 5a). Methylamine and ethylamine also can increase the radical emission of the **B-α-CD** system at a certain concentration range. However, the radical emission can be totally quenched by up to 50 μm concentrations of methylamine and ethylamine (Figure S21b,c). Moreover, adding methylamine and ethylamine in the system will lead to a longer photoactivated time of over 10 min to increase the radical emission (Figure S22). Hence, we can discern ammonia, methylamine, and ethylamine through emission intensity changes and distant photoactivated time. Interestingly, the free radical emission can be significantly quenched by aniline (Figure 5a,b). The ultraviolet–visible absorption spectrum of the **B-α-CD** system added with ammonia–water was also tested and showed radical absorption (Figure 5c), which indicated that the red-shift emission belongs to radical emission.

We also investigated the detection range of the ammonia–water free radical system (Figure 5d). The responding concentration is from 5 × 10^–6^ to 1.4 × 10^–4^ mol/L, and the detection response limit LOD is 1.82 × 10^–6^ mol (Figure 5e). In addition, other radical emission systems such as **B-TREH** and **B-SUC** also can be used to detect amine compounds, which showed a similar radical emission responding process after exposure to ammonia–water after light irradiation (Figure S23).

**Figure 6:**
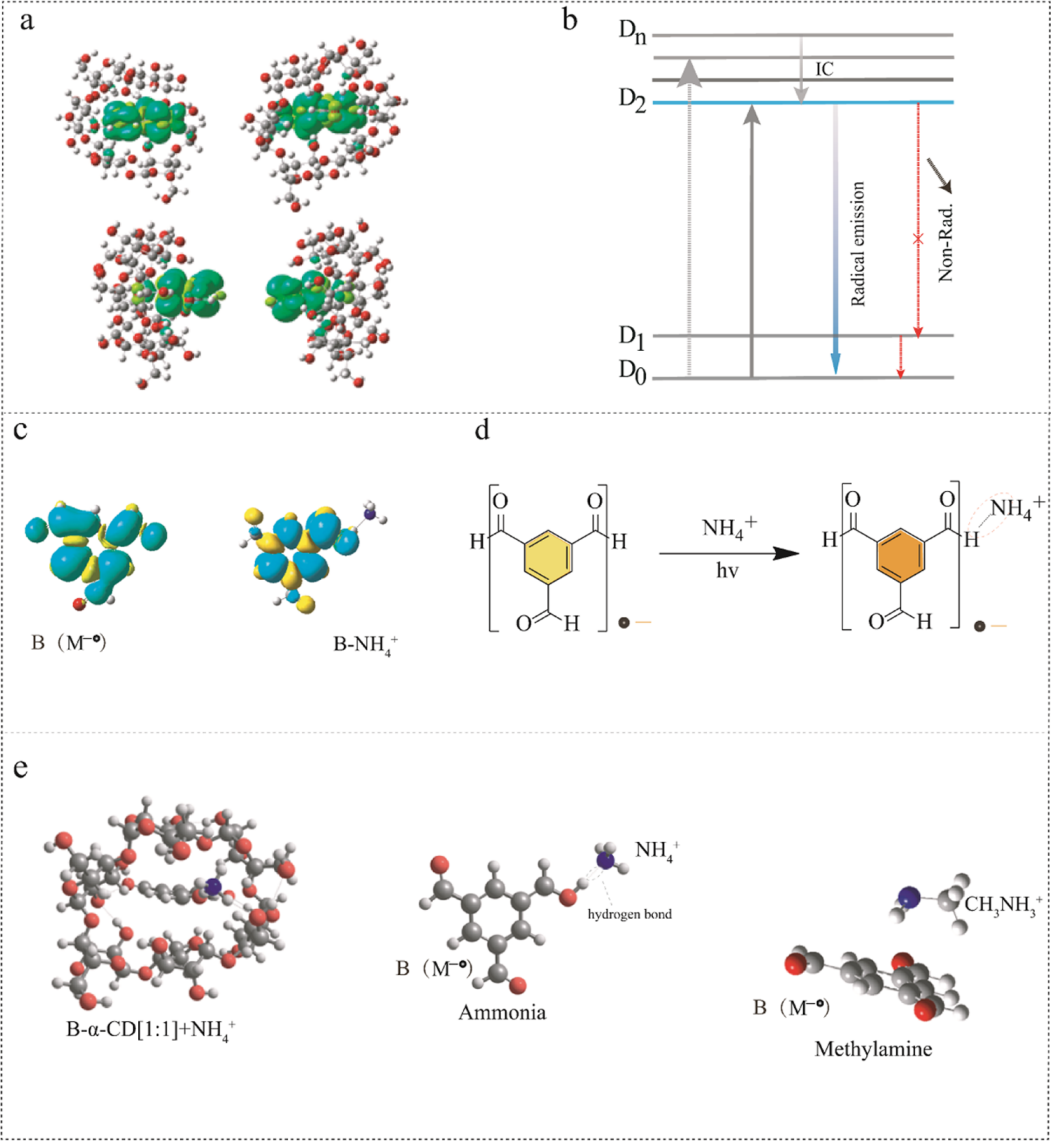
(a) Spin-density distribution of anion radical (M^–•^) for **B-α-CD** (complex [1:1]^−1^) in the gas phase in four different projections. (b) General emission mechanism of host–guest systems. (c) Spin-density map of compound **B** anion radical (M^–•^) and B-NH_4_^+^ (M^–•^ + NH_4_^+^). Blue or yellow regions indicate the areas of spin polarization. (d) Response process of ammonia with the **B** radical. (e) Optimized geometry after the addition of ammonia compounds.

For a better understanding of the photoinduced emission mechanism in the studied systems, quantum-chemical calculations were performed based on density functional theory (DFT). The carbonyl group of the guest molecule forms an obvious noncovalent bond with the hydroxyl group of the host molecule (Figure 6a). Meanwhile, the spin-density distribution of the anion radical in **B-α-CD** (complex [1:1]^−1^) is significantly delocalized (Figure 6a), which indicates the formation of stable free radicals in the system. In addition, time-dependent density functional theory (TD-DFT) simulations were performed (Tables S1–S5), and we have found that the internal quenching effect between the D_2_ and D_1_/D_0_ states is slow or weak because of the large energy gap between D_2_–D_1_ and D_2_–D_0_ (Tables S1–S5). This is similar to our previous study since the D_2_–D_0_ radiative transition itself is very strong and fast, and the anion radical of **B** was able to exhibit anti-Kasha emission from the D_2_ state (Figure 6b).^19^ Therefore, the weak interaction between host and guest molecules plays a crucial role in stabilizing the emission of free radicals. For that, a coassembly system was made by doping NH_3_ with **B-α-CD**. Experimental UV–vis spectra showed that the absorption became weak after adding ammonia after 365 nm (Figure 5c). The calculation results show that the fluorescence of photoinduced free radicals from this coassembly has no effect by doping the NH_3_ despite showing the weak blue shift in calculated absorbance results (Table S6). Based on these findings, we changed the NH_3_ species by ammonium (NH_4_^+^) cation and by the NH_3_·H_2_O complex. In our calculations, the energy and oscillator strength of the D_2_ state for the **B-α-CD** anion radical complex and the **B-α-CD-NH**_**4**_^**+**^ complex were obtained, respectively. (Table S7). **B-α-CD** anion radical, which had NH_4_^+^ or NH_3_·H_2_O, showed a red-shift of D_2_ state energy (at D_0_ ground state geometry) compared to the free **B-α-CD** anion radical (Figure 6c,d), which is in agreement with experimental observations: emission wavelength red-shifts from 550 nm without NH_3_·H_2_O to 575 nm with NH_3_·H_2_O.

Quantum-chemical calculations show that the strong stabilization of the overall assembly is due to the H-bonding (Figure 6c), and the internal quenching effect between D_2_ and D_1_/D_0_ states is slow or weak here due to large D_2_–D_1_ and D_2_–D_0_ energy gaps. According to our calculations, the spin-density maps of anion radicals are quite delocalized, and therefore, the systems are quite stable (Figure 6c–e). The optimized geometry of **B-α-CD-NH**_**4**_^**+**^ also shows that **B-NH**_**4**_^**+**^ enters the **α-CD** cavity that stabilizes the radical emission response (Figure 6e). However, other compounds like methylamine and ethylamine demonstrate no such strong H-bond effect for enhancing free radical emission. In the case of aniline, we observe the significant red-shift of the D_2_ state compared to the complex of compound **B** with NH_3_, which accelerates the internal conversion and thus quenches the emission in agreement with the experimental finding. In addition, we should note that the aliphatic amine molecule enhances the radical emission, but aromatic amine quenches it in a concentration range. In the case of the aliphatic amines possessing a lone pair of electrons on the N atom, they play the role of electron donor and form additional H-bonds with electron-deficient H atoms of matrix and dopant. Thus, it will further stabilize the free radicals and their luminescence. However, lone pairs of electrons on the nitrogen atom in aromatic amines (aniline) are in conjugation with the benzene ring, and their basic properties are very weak. At the same time, it is possible that aniline can form a charge-transfer complex with a radical dopant that quenches its luminescence.

## Conclusions

A host and guest coassembly strategy was used to obtain the free radical emission systems. The small hydroxyl molecules can transfer an electron to the carbonyl guest to promote the formation of free radicals and stabilize the excited state of carbonyl radicals based on intermolecular interactions. Importantly, carbonyl compounds could be assembled with more hydrophilic compounds (**α-CD**, trehalose, and sucrose, **F127**), which could induce the release of free radicals in water. Meanwhile, self-assembled aggregates exhibit different photoactive and temperature response behaviors. The NMR, absorption spectroscopy, and EPR spectroscopy were used to confirm the photoemission of free radicals. Furthermore, because carbonyl groups can interact with amino groups, cyclodextrin radical emission systems can be used to identify different ammonia compounds. The quantum-chemical calculations show the formation of stable complexes between the ammonia compound and the cyclodextrin system, where NH_4_^+^ forms a strong hydrogen bond with the **B-α-CD** complex where the **B** dopant is strongly coupled with the **α-CD** cavity. From the spin-density distribution of **B-α-CD** with NH_4_^+^, we conclude the significant delocalization of the unpaired electron not only over the **B** dopant but also over the **α-CD** host molecule. Therefore, the coassembly strategy is an efficient way to construct the free radical luminescent systems in different states that considerably broaden the application scope of free radicals.

## Experimental Section

### Preparation of Coassembly Solid Powder

Compound **A** or **B** (0.01, 0.1, 1, and 10 mg) with hydroxyl compounds (10 mg) in acetone (10 mL) were mixed in a round-bottomed flask, followed by sonication for 30 min. Then, acetone was slowly evaporated at room temperature to obtain the powder.

### Preparation of the B-α-CD Aqueous System

Different weights of **B** (0.1, 1, 3 mg, 5, 8 m, 10, and 20 mg) with **α-CD** (10 mg) were separately dissolved in an aqueous solution. Then, the solutions were sonicated to fully coassemble to obtain the **B-α-CD** system.

### Preparation of B–F127 NPs

One milligrams of **B** with **F127** (1 mg) was dissolved in THF (1 mL) and sonicated for 5 min. THF was evaporated by reduced pressure distillation. Then, the solid was in the water and sonicated for 5 min. The solutions were filtered through a 0.45 μm microfilter and collected the filtrate to obtain the **B–F127** NPs.

## Supporting Information Available

Materials, measurements, computational details, note I, note II, and references (PDF)
