# Supplementary material for: Photoinduced Carbonyl Radical Luminescence in Host–Guest Systems
Source: ACS Appl Mater Interfaces. 2023 Dec 12;15(50):58888–96. doi: 10.1021/acsami.3c14730 (PMC10739597; doi:10.1021/acsami.3c14730)
Supplement: Supplementary file 1 — am3c14730_si_001.pdf [file am3c14730_si_001.pdf]

Supporting Information

Photoinduced Carbonyl Radical Luminescence in Host-guest Systems

Juanjuan Liu,[†,#] Huajie Yu,[†,#] Farhan Siddique,[‡] Glib V. Baryshnikov,*[‡] Hongwei Wu*[†]

[†] Key Lab of Science and Technology of Eco-Textile, Ministry of Education, National Engineering Research Center for Dyeing and Finishing of Textiles, College of Chemistry, Chemistry and Chemical Engineering, Donghua University, Shanghai 201620 (China)

[‡] Laboratory of Organic Electronics, Department of Science and Technology, Linköping University, SE-60174 Norrköping, Sweden.

Corresponding Author

wuhongwei@dhu.edu.cn (Hongwei Wu)

glib.baryshnikov@liu.se (Glib V. Baryshnikov)

Materials

1,3,5-Triformylbenzene, 1,3,5-triacetylbenzene, trans-1,4-Cyclohexanedimethanol, 1,4-Benzenedimethanol, (1R)-1-phenylethane-1,2-diol, 4,4-Dimethylbiphenyl, 1,4-Dimethoxybenzene and α-Cyclodextrin were available from Bide Pharmatech Ltd. luronic® F-127 were available from Sigma AIdrich®. Trehalose was available from Shanghai yuan ye Bio-Technology Co., Ltd. 1,3,5-Benzenetrimethanol was available from Shanghai Macklin Biochemical Co., Ltd. D (+)-Sucrose and Ammonium solution was available from General-Reagent. Ethylamine solution and aniline were available from Adamas-beta®. Methylamine aqueous solution was available from Sinopharm Chemical ReagentCo., Ltd.

Measurements

^1H NMR spectra were measured on a Bruker 400 MHz spectrometer. Absorption spectra were recorded on a Shimadzu 2600 spectrophotometer, while the fluorescent emission was taken with a Fluoro Max spectrofluorometer and a Shimadzu RF-6000 fluorescence spectrophotometer. The fluorescent lifetimes were measured using a Fluoro Max spectrofluorometer with a time-correlated single photon counting technique. DLS measurements were made by Nano ZS. Powder X-ray diffraction (XRD) measurements were finished by SMART CCD from Bruker. EPR spectra were recorded on a Bruker ELEXSYS E500 spectrometer (300 K, film state), the microwave frequency is 9.617939. The photos were taken with a Cannon EOS-90D camera or Vivo X50. High-performance liquid chromatography (HPLC) data was obtained in Agilent Technologies Spectrometer with 1200 series.

Computational details

Compound **B** and its co-assembly with α-cyclodextrin were investigated theoretically. Optimization of the ground state geometries was performed with the density functional theory (DFT) method and B3LYP functional in a gas phase approximation[1, 2]. Spin-unrestricted DFT methodology for all calculations related to open-shell radical systems. The long-range corrected ωB97[3] and LC-ωHPBE[4] functionals were further applied for the excited state calculations using the time-dependent density functional theory (TDDFT)[5-7] and Tamm-Dancoff approximation (TDA)[8]. Environmental effects were comprised by means of the polarizable continuum model

(PCM)[9] using water as a solvent. For all the DFT calculations 6-311++g(d,p) basis set and the Gaussian16 program[10] suite were used.

Supplementary note I: Fluorescence, absorbance spectra, ^{1}H NMR spectra, HPLC analysis, Emission lifetime, and EPR spectra of the host, guest molecule, and free radical systems.

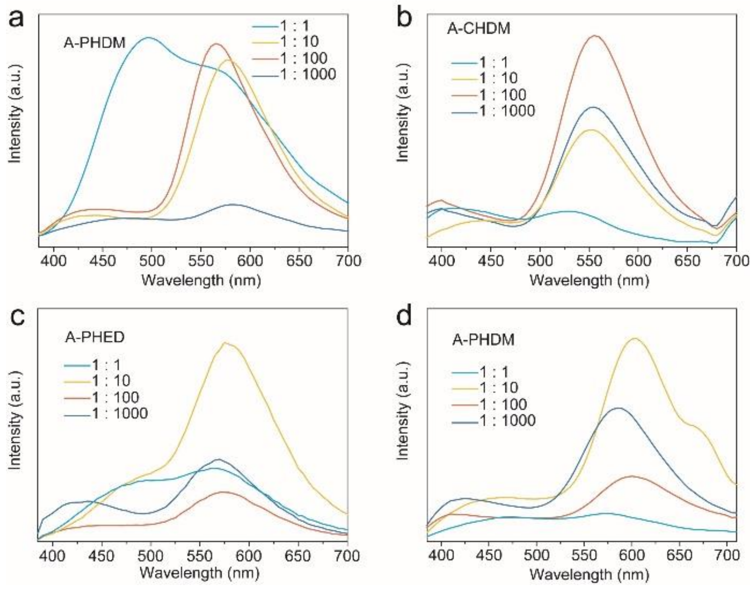

Figure S1. Fluorescence spectra of (a) **A-PHDM**, (b) **A-CHDM**, (c) **A-PHED**, and (d) **A-PHTM** at different doping concentrations after 365 nm light irradiation.

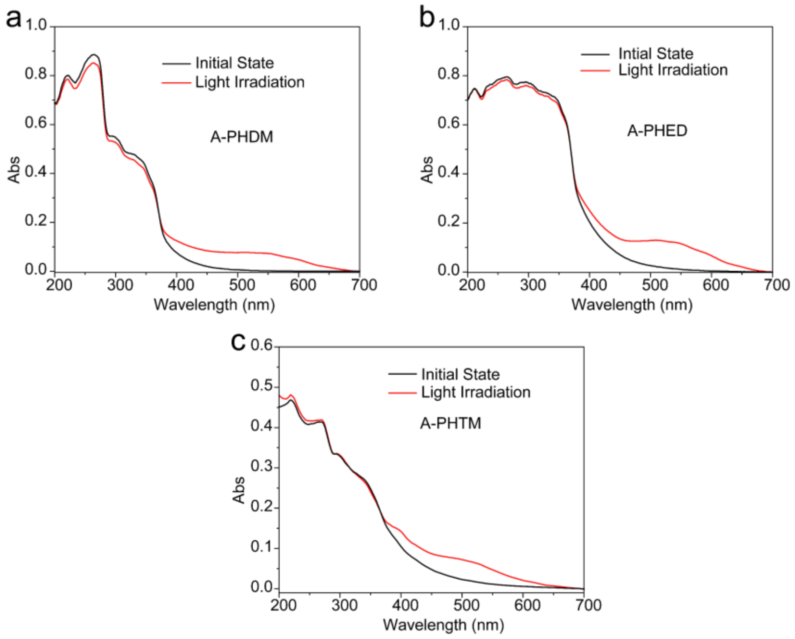

Figure S2. Absorbance spectra of (a) **A-PHDM**, (b) **A-PHED,** and (c) **A-PHTM** at the initial state and after 365 nm light irradiation.

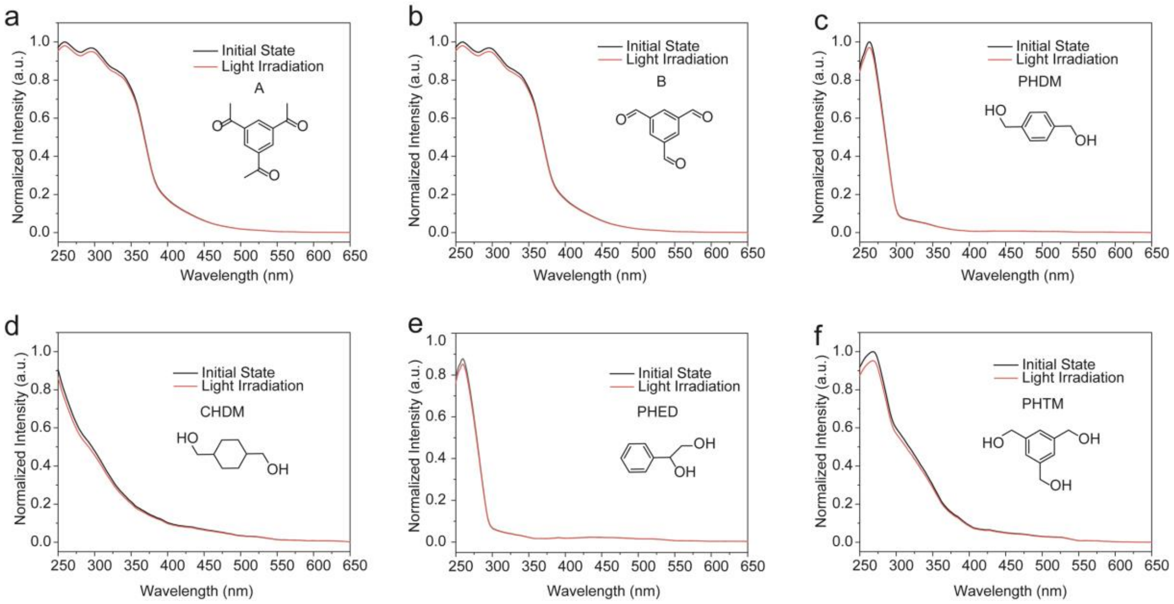

Figure S3. Absorbance spectra of (a) **A**, (b) **B**, (c) **PHDM**, (d) **CHDM**, (e) **PHED** and (f) **PHTM** before and after 365 nm light irradiation.

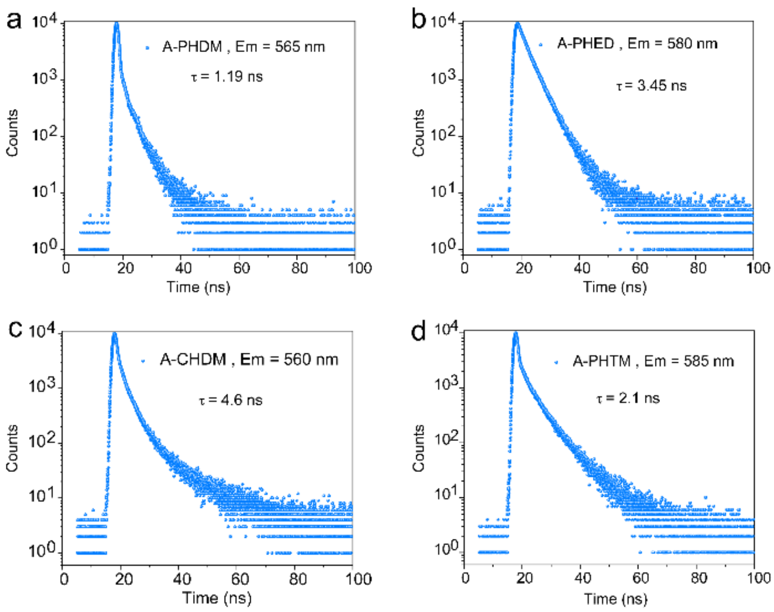

Figure S4. Emission lifetime of (a) **A-PHDM** (1: 100 wt %), (b) **A-PHED** (1: 10 wt %), (c) **A-CHDM** (1: 100 wt %), and (d) **A-PHTM** (1: 10 wt %) after 365 nm excitation. Em represents emission.

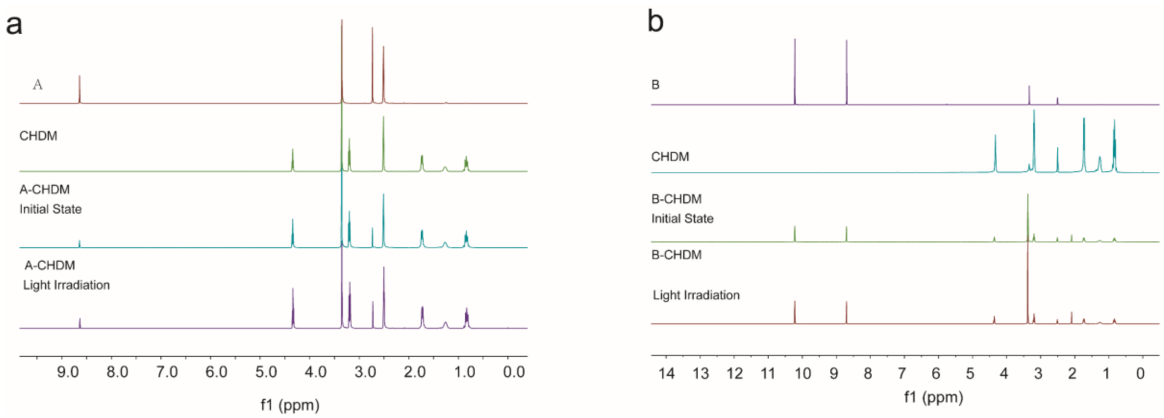

Figure S5. (a) ^{1}H NMR spectra of **A**, **CHDM**, and **A-CHDM** (1: 10 wt %) at the initial state and after 365 nm light irradiation in DMSO-d_6; (b) ^{1}H NMR spectra of **B**, **CHDM**, and **B-CHDM** (1: 10 wt %) at the initial state and after 365 nm light irradiation in DMSO-d_6.

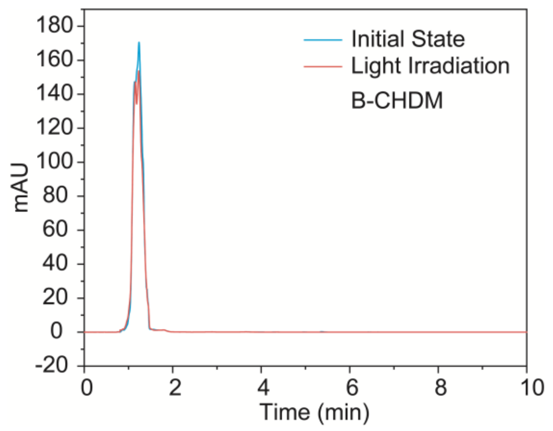

Figure S6. The HPLC analysis of B-CHDM (1:10) in acetonitrile /H_2O (7:3 by vol) mixture solution.

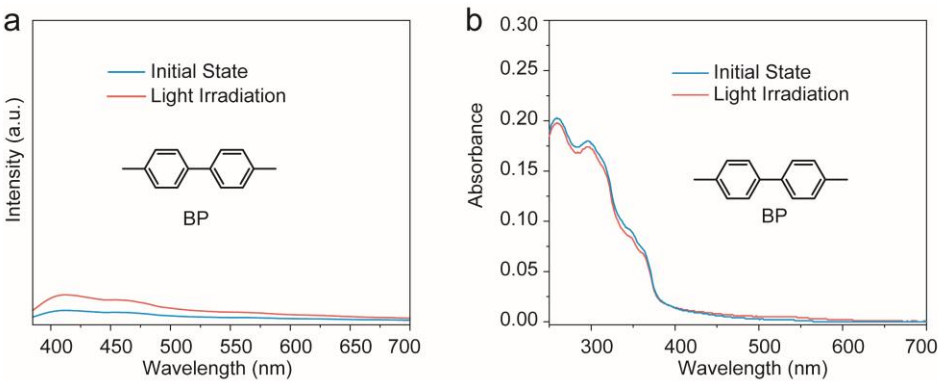

Figure S7. (a) Fluorescence and (b) Absorbance spectra of **BP** before and after light irradiation under the 365 nm excitation.

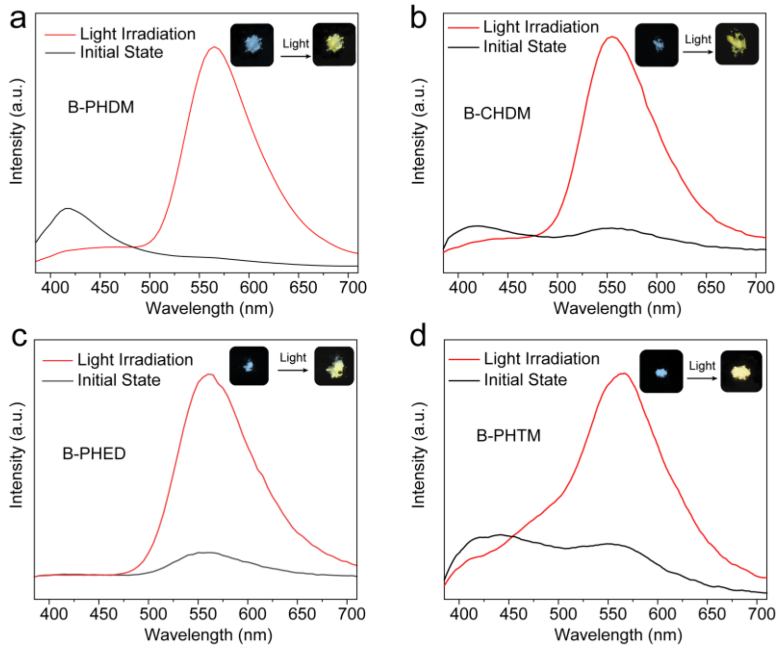

Figure S8. Fluorescence spectra of (a) **B-PHDM**, (b) **B-CHDM**, (c) **B-PHED**, and (d) **B-PHTM** at doping concentration (1: 100 wt %) after 365 nm light irradiation. We should note the weak yellow emission in spectra of the initial state powder is not the true emission of the initial powder, it should be due to the powder being too easy to be photoactivated by instrument excitation light in the test process.

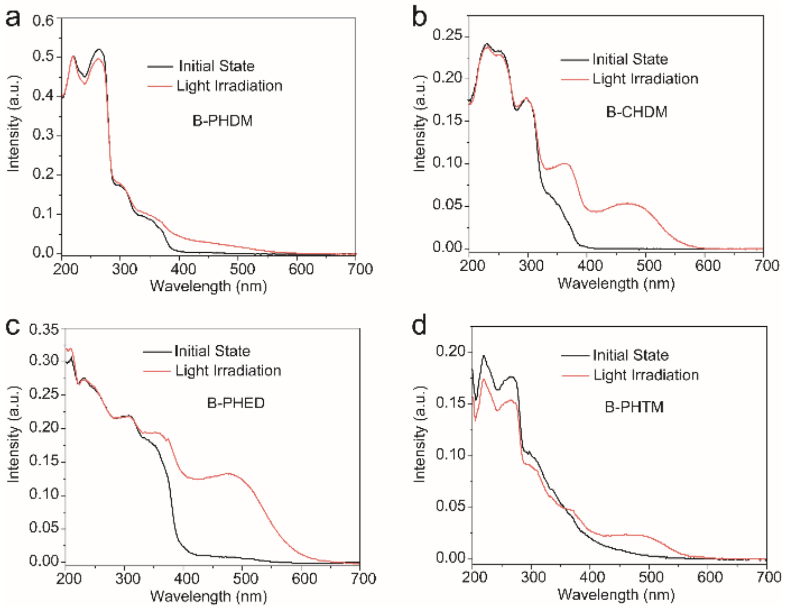

Figure S9. Absorbance spectra of (a) **B-PHDM**, (b) **B-CHDM**, (c) **B-PHED,** and (d) **B-PHTM** at the initial state and after 365 nm light irradiation.

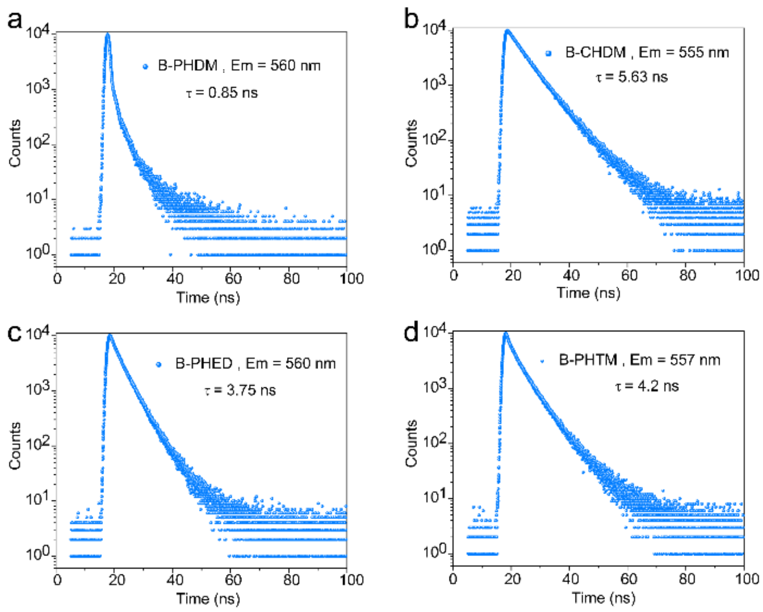

Figure S10. Emission lifetime of (a) **B-PHDM**, (b) **B-CHDM**, (c) **B-PHED**, and (d) **B-PHTM** (1: 100 wt %) under different emission and 365 nm excitation.

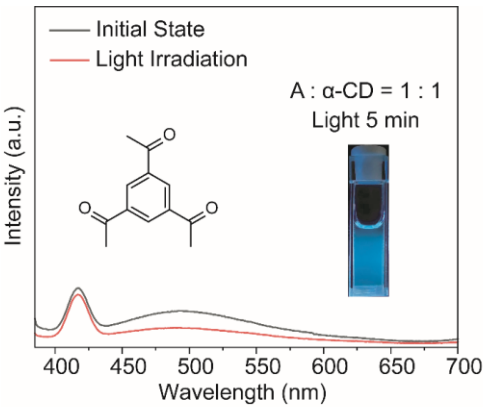

Figure S11. Emission spectra of **A-α-CD** (1: 1 wt %) after 365 nm light irradiation.

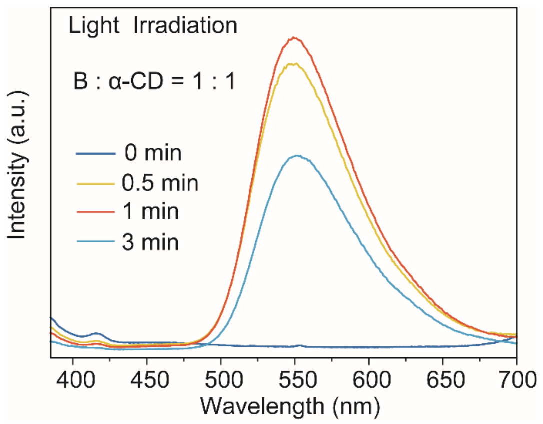

Figure S12. Emission spectra of **B-α-CD** (1: 1 wt %) after different light irradiation times.

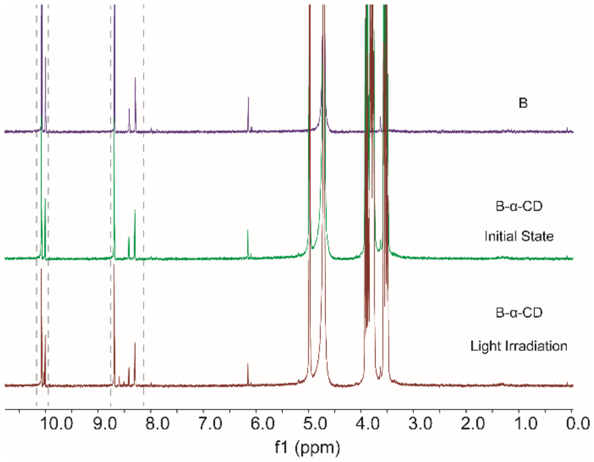

Figure S13. ^{1}H NMR spectra of **B**, **B-α-CD** (1: 10 wt %) at the initial state and after 365 nm light irradiation in D_2O. Multiple ^{1}H NMR peaks in the initial state of B in water are due to the B is easy to form the hydrates with water molecules according to the relevant literature.

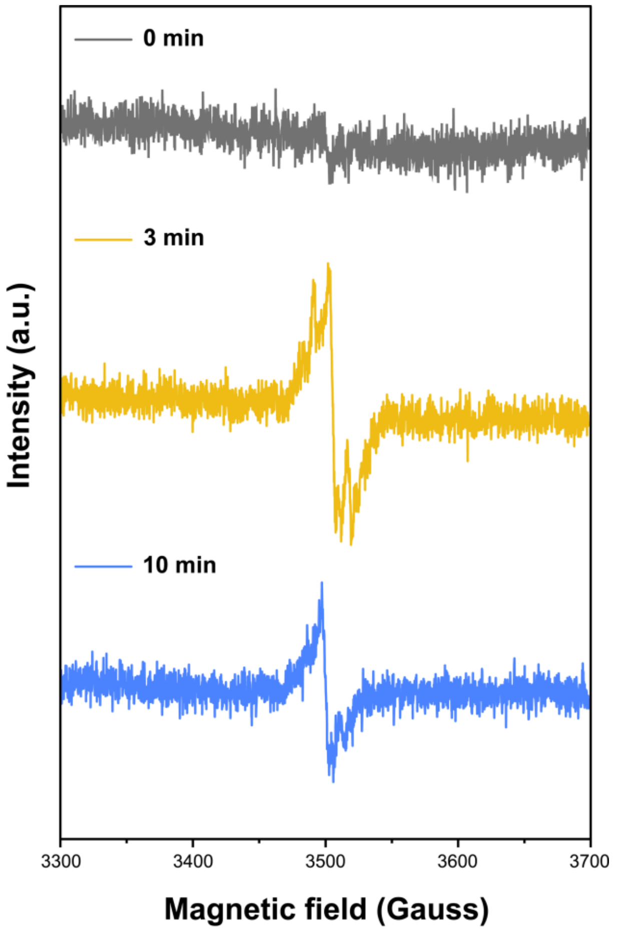

Figure S14. EPR spectra of **B-α-CD** (1: 1 wt %) after different light irradiation times.

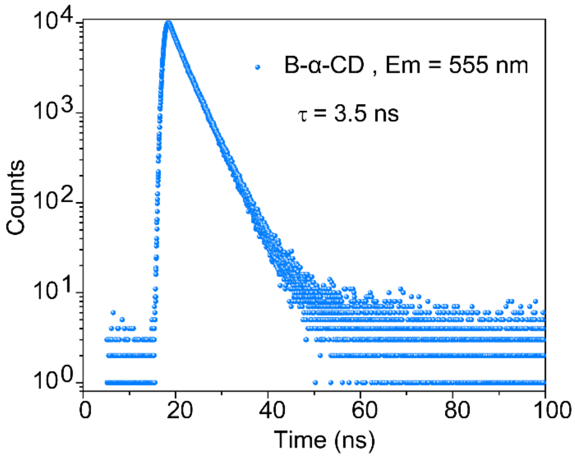

Figure S15. Emission lifetime of **B-α-CD** (1: 1 wt %) after 555 nm emission and 365 nm excitation.

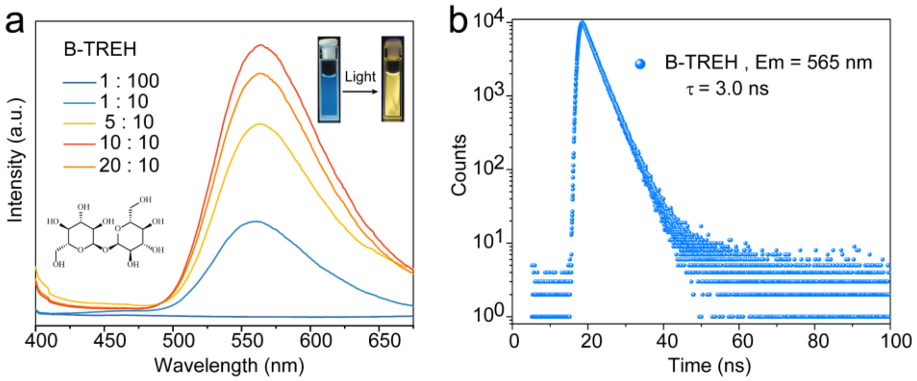

Figure S16. (a) Emission spectra of **B-TREH** at different doping concentrations and after 365 nm light irradiation and excitation. (b) Emission lifetime of **B-TREH** (1: 1 wt %) after 565 nm emission and 365 nm excitation.

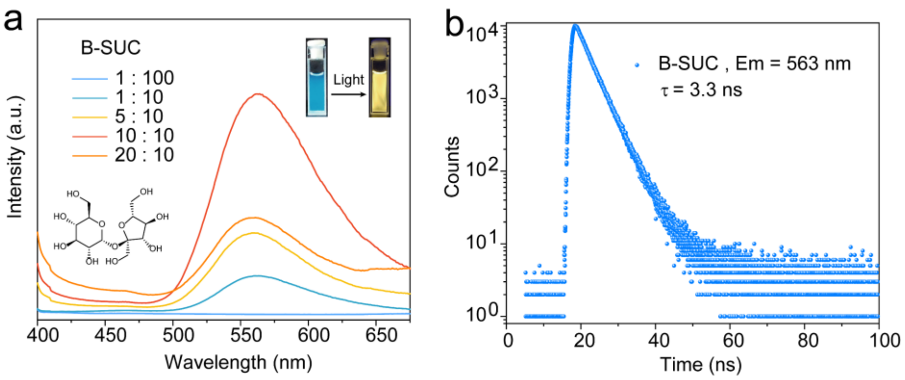

Figure S17. (a) Emission spectra of **B-SUC** at different doping concentrations after 365 nm light irradiation and excitation. (b) Emission lifetime of **B-SUC** (1: 1 wt %) after 563 nm emission and 365 nm excitation.

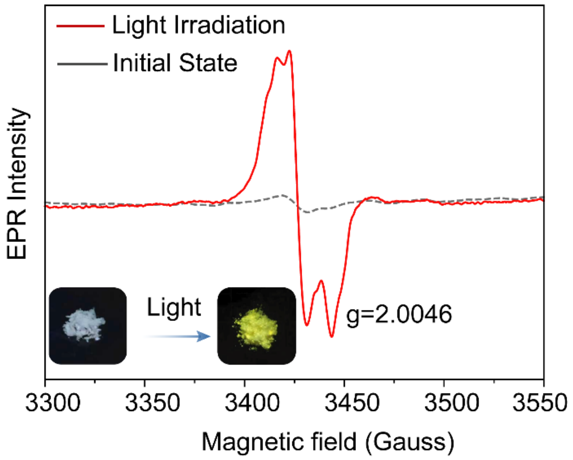

Figure S18. EPR spectra of **B-α-CD** (1: 1 wt %) in powder state.

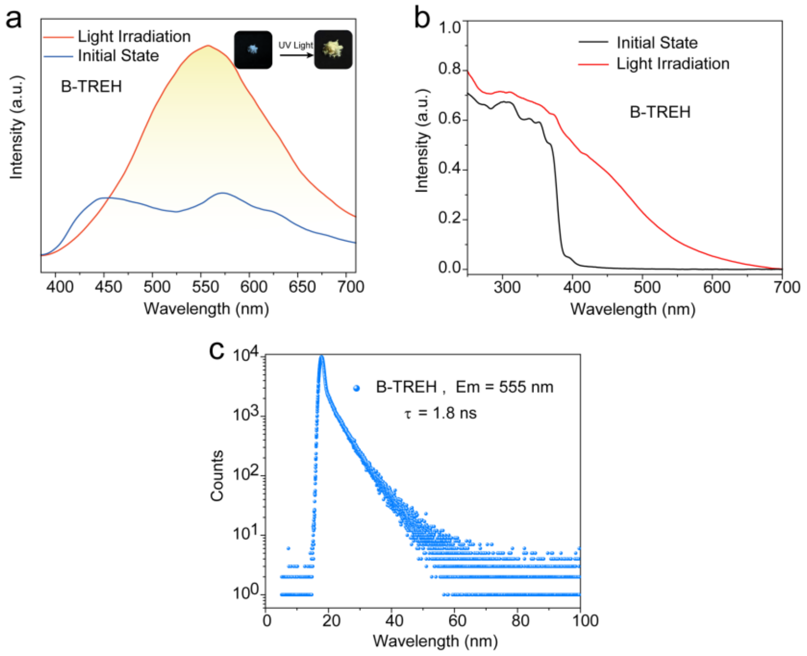

Figure S19. (a) Emission spectra of **B-TREH** (1: 1 wt %) at the initial state after 365 nm light irradiation and excitation. (b) Absorbance spectra of **B-TREH** at the initial state after 365 nm light irradiation. (c) Emission lifetime of **B-TREH** (1: 1 wt %) after 555 nm emission and 365 nm excitation.

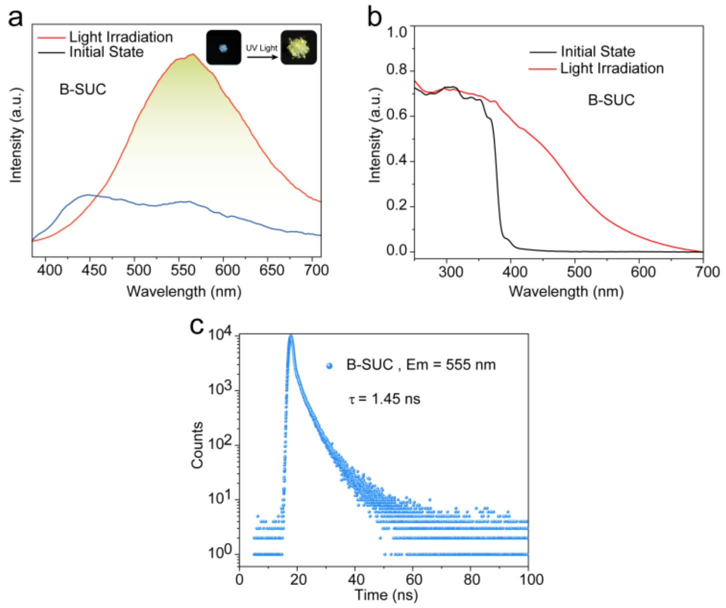

Figure S20. (a) Emission spectra of **B-SUC** (1: 1 wt %) at the initial state and after 365 nm light irradiation. (b) Absorbance spectra of **B-SUC** at the initial state and after 365 nm light irradiation. (c) Emission lifetime of **B-SUC** (1: 1 wt %) after 555 nm emission and 365 nm excitation. We should note the weak yellow emission in spectra of the initial state powder is not the true emission of the initial powder, it should be due to the powder being too easy to be photoactivated by instrument excitation light in the test process.

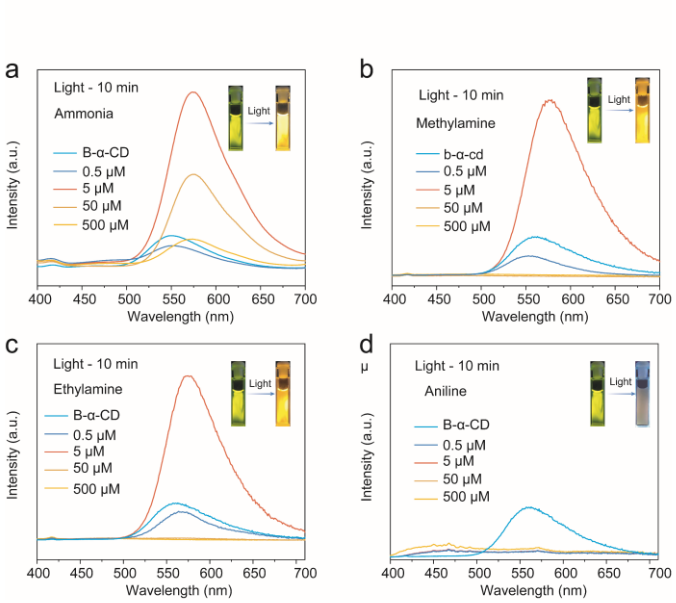

Figure S21. Fluorescence emission spectra of **B-α-CD** solutions after the addition of different concentrations of (a) Ammonia, (b) Methylamine, (c) Ethylamine, and (d) Aniline, respectively.

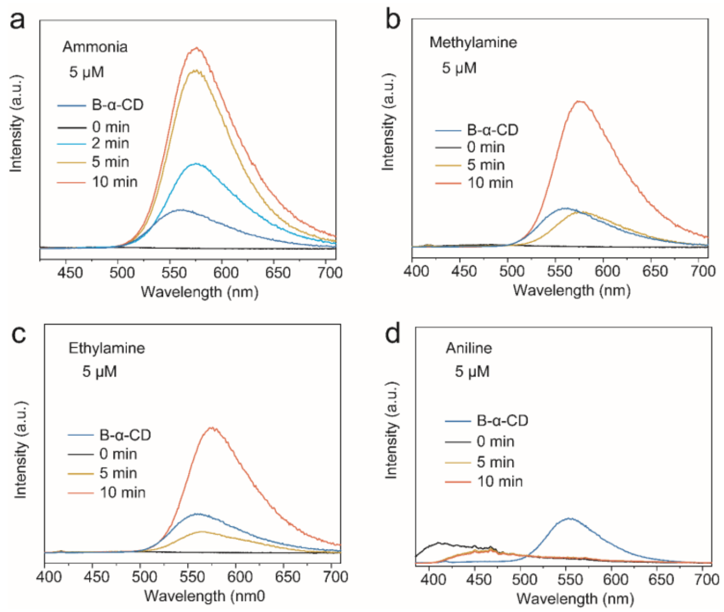

Figure S22. Fluorescence emission spectra of **B-α-CD** solutions after the addition of (a) Ammonia, (b) Methylamine, (c) Ethylamine, and (d) Aniline (5 μM) under various irradiation times, respectively.

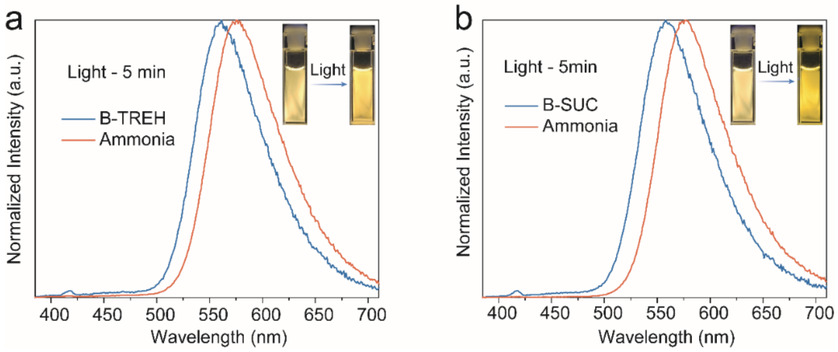

Figure S23. (a) Fluorescence emission spectra of B-TREH and after the addition of Ammonia in it after 365 nm light irradiation. (b) Fluorescence emission spectra of B-SUC and after the addition of Ammonia in it after 365 nm light irradiation.

Supplementary note II: Theoretical calculation data for host, guest molecule, and free radical systems.

Table S1. DFT/TD/wb97xd/6-311++g(d,p) in the gas phase.

	B^{-1}		Complex $[1:1]^{-1}$	
Excited State	Energy (nm)	Osc.str. *(f)*	Energy (nm)	Osc.str. *(f)*
D_1	2145	0.005	1181	0.000
D_2	832	0.117	669	0.090
D_3	527	0.000	371	0.000
D_4	452	0.001	365	0.000
D_5	396	0.000	356	0.007
D_6	377	0.006	355	0.001
D_7	357	0.005	316	0.002
D_8	356	0.000	315	0.005
D_9	351	0.021	311	0.000
D_{10}	344	0.000	309	0.012

Table S2. DFT/TDA/wb97xd/6-311++g(d,p) in the gas phase.

	B^{-1}		Complex $[1:1]^{-1}$	
Excited State	Energy (nm)	Osc.str. *(f)*	Energy (nm)	Osc.str. *(f)*
D_1	1795	0.005	1080	0.000
D_2	734	0.141	615	0.109
D_3	527	0.000	371	0.000
D_4	452	0.001	360	0.000
D_5	396	0.000	351	0.000
D_6	377	0.006	330	0.006
D_7	351	0.000	315	0.005
D_8	349	0.026	314	0.002
D_9	340	0.000	309	0.000
D_{10}	340	0.000	307	0.000

Table S3. DFT/TD/LC-wHPBE/6-311++g(d,p) in the gas phase.

	B^{-1}		Complex $[1:1]^{-1}$	
Excited State	Energy (nm)	Osc.str. *(f)*	Energy (nm)	Osc.str. *(f)*
D_1	1772	0.008	881	0.001

D_2	771	0.153	553	0.128
D_3	467	0.000	374	0.000
D_4	402	0.001	364	0.000
D_5	364	0.000	361	0.009
D_6	363	0.002	328	0.000
D_7	359	0.000	304	0.000
D_8	344	0.000	303	0.000
D_9	341	0.006	294	0.000
D_{10}	334	0.000	285	0.027

Table S4. DFT/TDA/LC-wHPBE/6-311++g(d,p) in the gas phase.

	B^{-1}		**Complex $[1:1]^{-1}$**	
Excited State	Energy (nm)	Osc.str. *(f)*	Energy (nm)	Osc.str. *(f)*
D_1	1400	0.006	796	0.000
D_2	658	0.162	507	0.139
D_3	466	0.000	367	0.000
D_4	402	0.001	358	0.000
D_5	359	0.000	328	0.000
D_6	358	0.000	321	0.006
D_7	341	0.006	302	0.000
D_8	339	0.000	301	0.000
D_9	330	0.000	295	0.001
D_{10}	328	0.006	285	0.029

Table S5. Complex $[CD:1]^{-1}$ comparison using GAS and PCM (solvent= water) model in gaussian.

	**Complex $[CD:B]^{-1}$**			**Complex $[CD:B]^{-1}$**		
	**TD-wb97xd**	**Gas**		**TD-wb97xd**	**PCM**	
**Excited State**	**Energy (eV)**	**Energy (nm)**	**Osc.str. *(f)***	**Energy (eV)**	**Energy (nm)**	**Osc.str. *(f)***
D_1	1.05	1181	0.000	1.00	1240	0.001
D_2	1.85	669	0.090	1.71	726	0.122
D_3	3.34	371	0.000	3.46	358	0.000
D_4	3.40	365	0.000	3.50	354	0.009
D_5	3.48	356	0.007	3.64	341	0.000
D_6	3.49	355	0.001	3.74	331	0.000
D_7	3.92	316	0.002	3.96	313	0.001
D_8	3.93	315	0.005	4.02	308	0.019
D_9	3.98	311	0.000	4.04	307	0.000

D_{10}	4.01	309	0.012	4.13	300	0.000
	**TDA-wb97xd**	**Gas**		**TDA-wb97xd**	**PCM**	
D_1	1.15	1080	0.000	1.10	1127	0.000
D_2	2.02	615	0.109	1.88	661	0.136
D_3	3.34	371	0.000	3.51	354	0.000
D_4	3.44	360	0.000	3.68	337	0.000
D_5	3.54	351	0.000	3.75	331	0.000
D_6	3.75	330	0.006	3.76	330	0.007
D_7	3.94	315	0.005	3.99	311	0.000
D_8	3.95	314	0.002	4.07	305	0.000
D_9	4.01	309	0.000	4.10	302	0.019
D_{10}	4.04	307	0.000	4.16	298	0.000
	**TD/LC-wHPBE**	**Gas**		**TD/LC-wHPBE**	**PCM**	
D_1	1.41	881	0.001	1.36	910	0.002
D_2	2.24	553	0.128	2.11	587	0.168
D_3	3.32	374	0.000	3.37	368	0.000
D_4	3.40	364	0.000	3.46	359	0.010
D_5	3.43	361	0.009	3.54	350	0.000
D_6	3.78	328	0.000	4.12	301	0.000
D_7	4.08	304	0.000	4.17	298	0.000
D_8	4.09	303	0.000	4.19	296	0.001
D_9	4.17	297	0.001	4.23	293	0.018
D_{10}	4.21	295	0.024	4.23	293	0.015
**D_n-D_0** emission based on TDA/LC-wHPBE calculations.						
	**TDA/LC-wHPBE**	**Gas**		**TDA/LC-wHPBE**	**PCM**	
D_1	1.56	796	0.000	1.52	816	0.001
D_2	**2.45**	**507**	**0.139**	**2.32**	**534**	**0.169**
D_3	3.38	367	0.000	3.43	361	0.000
D_4	3.46	358	0.000	3.60	345	0.000
D_5	3.79	328	0.000	3.88	320	0.006
D_6	3.87	321	0.006	4.16	298	0.000
D_7	4.11	302	0.000	4.17	297	0.000
D_8	4.12	301	0.000	4.22	294	0.000
D_9	4.20	295	0.001	4.26	291	0.000
D_{10}	4.34	285	0.029	4.36	284	0.037

Table S6. Dn-D_0 vertical emission of anion radical (**B–•**) species for **B-α-CD**（NH_3 is the model）

based on TDA/LC-wHPBE calculations.

	Complex $[1:1]^{-1} + NH_3$			Complex $[1:1]^{-1} + NH_3$		
	TDA/LC-wHPBE	Gas		TDA/LC-wHPBE	PCM	
D_1	1.59	781	0.001	1.55	800	0.003
D_2	2.51	495	0.139	2.37	523	0.170
D_3	3.45	359	0.001	3.46	359	0.001
D_4	3.51	353	0.000	3.65	340	0.000
D_5	3.82	324	0.000	3.88	320	0.006
D_6	3.87	321	0.005	4.16	298	0.000
D_7	4.16	298	0.000	4.18	296	0.000
D_8	4.18	297	0.000	4.25	292	0.001
D_9	4.22	294	0.001	4.28	290	0.000
D_{10}	4.37	284	0.028	4.39	282	0.038

*Note：Accounting for water solvent within polarizable continuum model (PCM) shifts D_2 energy slightly to lower energy comparing to gas phase results for any type of studied systems. We should note, that the size of system doesn't allow to optimize the structure of D_2 excited state and predict the fluorescence wavelength, but we assume that synchronous Stocks shift will just shift the relaxed D_2 state energy to the red region, but not lead to the new qualitative results.

Table S7. Dn-D_0 vertical emission of anion radical (**B–•**) species for **B-α-CD**（NH_4^+ and $NH_3{\cdot}H_2O$ were used as models）based on TDA/LC-wHPBE calculations.

	Complex $[1:1]^{-1} + NH_4$			Complex $[1:1]^{-1} + NH_3 + H_2O$		
	TDA/LC-wHPBE	Gas		TDA/LC-wHPBE	Gas	
D_1	1.22	1012	0.015	1.46	850	0.001
D_2	2.20	565	0.133	2.33	532	0.138
D_3	3.50	354	0.000	3.44	361	0.000
D_4	3.81	325	0.001	3.53	351	0.000
D_5	3.89	319	0.000	3.81	326	0.000
D_6	4.11	302	0.000	3.85	322	0.002
D_7	4.17	297	0.000	4.09	303	0.000
D_8	4.23	293	0.024	4.21	295	0.000
D_9	4.27	291	0.005	4.25	291	0.001
D_{10}	4.51	275	0.009	4.33	286	0.034

Table S8. Optimized geometries, Energies (a.u.) and Spin density**.**

SYSTEM	Optimized Geom	S_0-optimized Energy (a.u.)	Spin Density
**B**	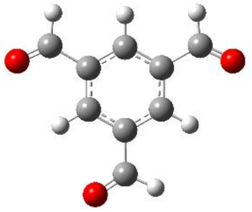	-572.3924212	
**B^{-1}**	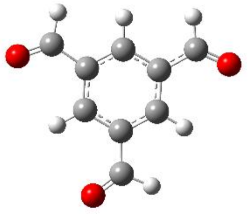	-572.452862	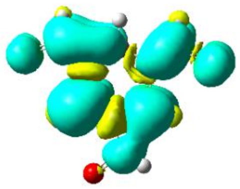
**$B\text{-}NH_3^{-1}$**	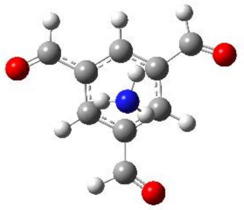	-629.045000	
**$B\text{-}NH_4^{+}$** **(Gas phase not worked)**	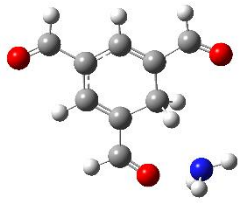	-629.5541263<br><br>(Not correct in the gas phase)	
**$B\text{-}NH_4^{+}$** **(Using PCM method solvent water here)**	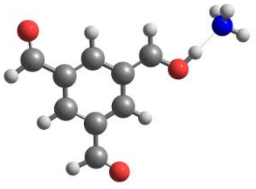	-629.589308	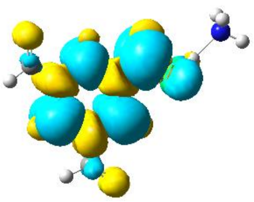
**α-CD Neutral**	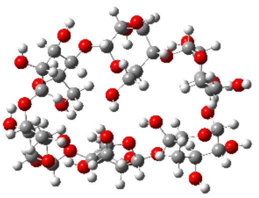	-3665.786893	

**B-α-CD-Complex $[1:1]^{-1}$**	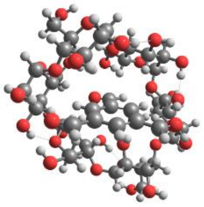	-4238.363768	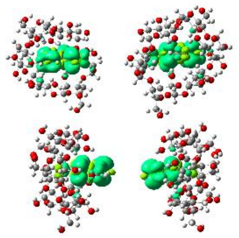
**B-α-CD-Complex-$NH_3[1:1]^{-1}$**	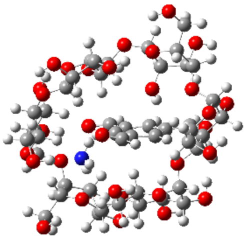	-4294.960617	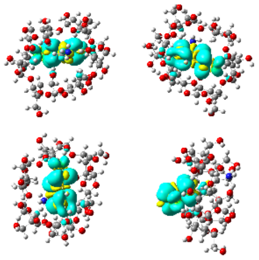
**B-α-CD-Complex [1:1] + NH_4**	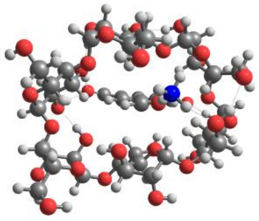	-4295.467349	
**B-α-CD-Complex $[1:1]^{-1}$ + NH_3 + H_2O**	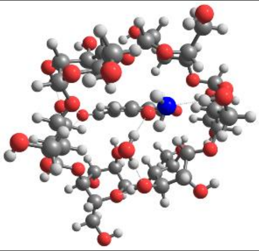	-4371.444814	
**B-Aniline**	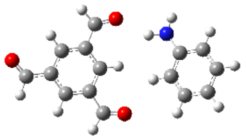	-860.164604	
**B-ethylamine**	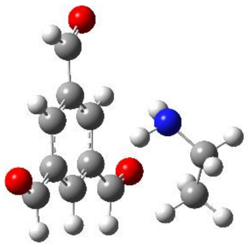	-707.690085	

References

S1. Becke, A. D. Density-Functional Thermochemistry. III. the Role of Exact Exchange. *J. Chem. Phys.* **1993**, *98* (7), 5648-5652.

S2. Lee, C.; Yang, W.; Parr, R. G. Development of the Colle-Salvetti Correlation-Energy Formula into a Functional of the Electron Density. *Phys. Rev. B.* **1988**, *37* (2), 785-789.

S3. Bauernschmitt, R.; Ahlrichs, R. Treatment of Electronic Excitations within the Adiabatic Approximation of Time Dependent Density Functional Theory. *Chem. Phys. Lett.* **1996**, *256* (4-5), 454-464.

S4. Henderson, T. M.; Izmaylov, A. F.; Scalmani, G.; Scuseria, G. E. Can Short-Range Hybrids Describe Long-Range-Dependent Properties? *J. Chem. Phys.* **2009**, *131* (4), 044108.

S5. Casida, M. E. Time-Dependent Density Functional Response Theory for Molecules. *Recent Adv. Dens. Funct. Methods* **1995**, 155-192.

S6. Furche, F.; Ahlrichs, R. Erratum: Adiabatic Time-Dependent Density Functional Methods for Excited State Properties. *J. Chem. Phys.* **2002**, *117*, 7433–7447.

S7. Runge, E.; Gross, E. K. U. Density-Functional Theory for Time-Dependent Systems. *Phys. Rev. Lett.* **1984**, *52* (12), 997-1000.

S8. Hirata, S.; Head-Gordon, M. Time-Dependent Density Functional Theory within the Tamm-Dancoff Approximation. *Chem. Phys. Lett.* **1999**, *314* (3-4), 291-299.

S9. Tomasi, J.; Mennucci, B.; Cammi, R. Quantum Mechanical Continuum Solvation Models. *Chem. Rev.* **2005**, *105* (8), 2999-3094.

S10. Bloino, J.; Baiardi, A.; Biczysko, M. Aiming at an Accurate Prediction of Vibrational and Electronic Spectra for Medium-to-Large Molecules: An Overview. *Int. J. Quantum Chem.* **2016**,

116 (21), 1543-1574.
